# AI in Cardiology: Improving Outcomes for All

**DOI:** 10.1016/j.jacadv.2024.101229

**Published:** 2024-09-25

**Authors:** Faraz S. Ahmad, Sadeer G. Al-Kindi, Steve Steinhubl

**Figure:**
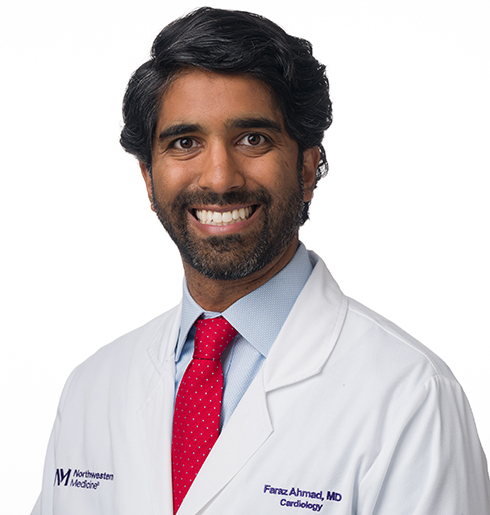

**Figure:**
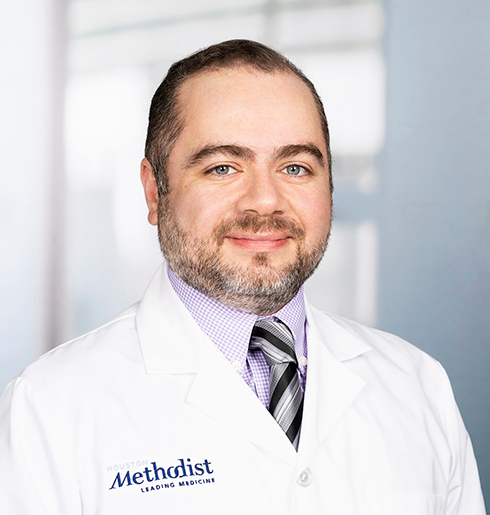

**Figure:**
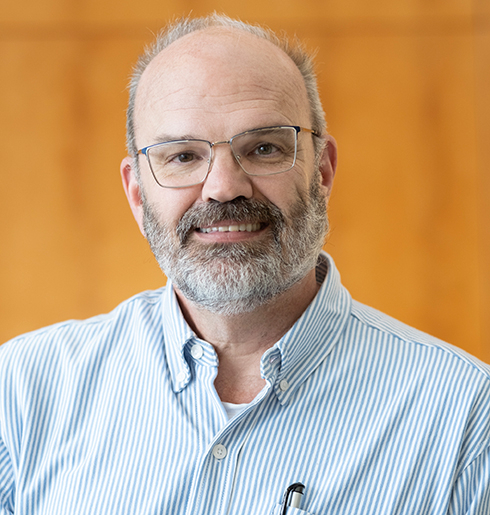

We are thrilled to publish the special issue, “AI in Cardiology: Improving Outcomes for All,” in *JACC: Advances*. The mission of *JACC: Advances* includes showcasing emerging areas of science that will alter the practice of cardiovascular medicine. The November 2022 public release of ChatGPT simultaneously increased public accessibility to artificial intelligence (AI) and powerfully demonstrated its potential impact of a broad set of tasks across nearly every industry and our personal lives. Undoubtedly, the use and impact of AI in cardiovascular medicine will grow over time. However, many questions remain about the scope of its impact in current practice and how we move from the immense enthusiasm for AI to improving cardiovascular care and, ultimately, equitable outcomes.

This issue compiles 20 original research articles, a brief report, and research letters that cover a broad range of AI applications and cardiovascular conditions, highlighting the how expansive the potential for AI will likely be in the future of cardiovascular care. Several articles train and test new AI models to tackle diverse problems. These include using computer vision and cardiac imaging to automate measurement of coronary artery disease, left ventricular ejection fraction, and right atrial pressures.^1^, ^2^, ^3^ Additional computer vision studies extract features associated with adverse outcomes from the liver and epicardial adipose tissue from cardiac computed tomography imaging.^4^^,^^5^ Consistent with the growing interest using multimodal data, another paper integrates echocardiogram and electrocardiogram data to model right ventricular function.^6^ Other articles use AI models for screening for familial hypercholesteremia, evaluating for heart disease in pediatric patients, or better phenotyping and risk stratification of various conditions and risk factors, including acute coronary syndromes, aortic stenosis, heart failure, incident cardiovascular disease, hyperkalemia, and social isolation.^7^, ^8^, ^9^, ^10^, ^11^, ^12^, ^13^, ^14^, ^15^, ^16^ Another study analyzes public perception of glucagon-like peptide 1 receptor agonists using natural language processing of social media posts.^17^

This special issue also highlights some of the methodological challenges when training AI models. Using a convolutional neural network on electrocardiogram data for the identification of patients with cardiac amyloidosis, Vrudhula et al demonstrate the importance of having accurate labels and diverse controls when training models.^18^ As AI models move from the research realm to clinical practice, more studies that examine methodological areas in training as well as best practices for validation, including ongoing monitoring and governance after deployment, are needed.

This collection includes a secondary analysis of a randomized controlled trial of patients with type 2 diabetes and hypertension. This study shows that a digital twin-based intervention that promoted medication adherence and healthy behaviors was associated with improvements in blood pressure control and other hypertension-related measures.^19^

Despite the promise of early research, prospective studies evaluating AI-enabled technologies remain rare compared with retrospective analyses describing model development and validation. The scoping review by Moosvi et al summarizes cardiovascular AI interventions that have been prospectively validated and highlights the limited number of these trials and low percentage that have made their data or models publicly available.^20^

The original research articles, brief report, and research letters are complemented by a State-of-the-Art Review on the role of AI in the management of syncope, providing a detailed overview of a patient-centric use case of a variety of AI tools.^21^ In addition, 3 Viewpoints tackle several key areas of consideration as AI implementation expands including the prominent role of venture capital firms in the cardiovascular digital health landscape, the use of multimodal, large language models, and the potential and challenges of using AI-enabled technologies to advance health equity.^22^, ^23^, ^24^

Taken together, this collection of articles covers wide range of conditions, data types, modeling approaches, and methodological and implementation issues related to AI and cardiovascular medicine. One of the key takeaways from the collection is that we are still early in use of AI for improving outcomes in cardiovascular medicine. Although model development and validation with retrospective data continues to progress at a torrential pace, so much more work is needed related to implementation, including more pragmatic clinical trials, studies that leverage approaches from the fields of human-computer interaction and implementation science, and the development of best practices for health systems when evaluating clinical effectiveness. The risks of AI to perpetuate bias and inequities and negatively impact patient privacy and data security remain high. Research to understand and address these risks as well as the many unique ethical concerns associated with the changing clinician-patient dynamic AI will bring are needed.

Since the founding of the field of AI at the Dartmouth Summer Research Project on Artificial Intelligence in 1956, the discipline has undergone boom and bust cycles, referred to as AI summers and winters. Certainly, since the launch of ChatGPT, it feels like a very hot summer. However, while the current exuberance for AI in medicine likely overestimates its short-term impact on clinical care, in the long term, it is poised to revolutionize how health care is delivered. Achieving our communities’ ultimate goal of improving cardiovascular outcomes will require substantial investment and rigorous research. At *JACC: Advances*, we intend to continue to lead in the publication of AI research that advances our field, especially pertaining to implementation of these technologies into clinical care.

## Funding support and author disclosures

Dr Ahmad reported that he has received research support from 10.13039/100004319Pfizer Inc and Atman Health. The other authors have reported that they have no relationships relevant to the contents of this paper to disclose.
